# Reference proteomes of five wheat species as starting point for future design of cultivars with lower allergenic potential

**DOI:** 10.1038/s41538-023-00188-0

**Published:** 2023-03-25

**Authors:** Muhammad Afzal, Malte Sielaff, Ute Distler, Detlef Schuppan, Stefan Tenzer, C. Friedrich H. Longin

**Affiliations:** 1 State Plant Breeding Institute, University of Hohenheim, Fruwirthstr. 21, 70599 Stuttgart, Germany; 2 Institute for Immunology and Research Center for Immune Therapy (FZI), University Medical Center of the Johannes Gutenberg University Mainz, Langenbeckstr. 1, 55131 Mainz, Germany; 3 Institute of Translational Immunology and Research Center for Immune Therapy (FZI), University Medical Center of the Johannes Gutenberg University Mainz, Langenbeckstr. 1, 55131 Mainz, Germany; 4 Division of Gastroenterology, Beth Israel Deaconess Medical Center, Harvard Medical School, 330 Brookline Avenue, Boston, MA 02215 USA

**Keywords:** Plant sciences, Proteomics

## Abstract

Wheat is an important staple food and its processing quality is largely driven by proteins. However, there is a sizable number of people with inflammatory reactions to wheat proteins, namely celiac disease, wheat allergy and the syndrome of non-celiac wheat sensitivity. Thus, proteome profiles should be of high importance for stakeholders along the wheat supply chain. We applied liquid chromatography-tandem mass spectrometry-based proteomics to establish the flour reference proteome for five wheat species, ancient to modern, each based on 10 cultivars grown in three diverse environments. We identified at least 2540 proteins in each species and a cluster analyses clearly separated the species based on their proteome profiles. Even more, >50% of proteins significantly differed between species - many of them implicated in products’ quality, grain-starch synthesis, plant stress regulation and proven or potential allergic reactions in humans. Notably, the expression of several important wheat proteins was found to be mainly driven by genetics vs. environmental factors, which enables selection and refinement of improved cultivars for the wheat supply chain as long as rapid test methods will be developed. Especially einkorn expressed 5.4 and 7.2-fold lower quantities of potential allergens and immunogenic amylase trypsin inhibitors, respectively, than common wheat, whereas potential allergen content was intermediate in tetraploid wheat species. This urgently warrants well-targeted clinical studies, where the developed reference proteomes will help to design representative test diets.

## Introduction

Wheat is one of the most important staple foods with a worldwide production of 765 million tons in 2019 (https://www.fao.org/faostat/en/#data/QCL, accessed on 08.12.2021) and provides 20% of the daily intake of dietary protein together with fiber, minerals and vitamins^1^. Most of the production is contributed by modern species common wheat (*Triticum aestivum* ssp. *aestivum*) and durum (*Triticum turgidum* ssp. *durum*). While common wheat is cultivated globally on almost 223 million hectares (https://apps.fas.usda.gov/psdonline/app/index.html#/app/advQuery, accessed on 29.01.2022) for bread production and animal nutrition, durum wheat covers worldwide 16 million hectares primarily for pasta production^2^. Although, ancient species spelt (*Triticum aestivum* ssp. *spelta*), emmer (*Triticum turgidum* ssp. *dicoccum*) and einkorn (*Triticum monococcum* ssp. *monococcum*) have been utilized as food for thousands of years^3–5^, they are currently cultivated only on a small-scale confined to specific regions^6–8^. Wheat grains contain roughly 8–15% protein of dry weight^9^, which can be classified into albumins/globulins (15–20%), including important essential amino acids^10^, and gluten proteins (80–85%)^9,11^. The viscoelastic and gustatory attributes as hallmarks of the quality for bread and pasta production are mainly endowed by the gluten proteins^12,13^. Simultaneously, some wheat proteins can trigger inflammatory reactions such as celiac disease (CeD), classical wheat allergy (WA), and non-celiac wheat sensitivity (NCWS) in approximately 1%, below 1%, and up to 10% of the wheat-consuming populations, respectively^14^. Specific gluten peptide sequences cause CeD^15,16^, alpha-amylase/trypsin inhibitors (ATIs) stimulate innate immune cells via toll-like receptor 4 (TLR4) to promote intestinal and extraintestinal inflammation in animal models of disease^17–23^, and especially serpins, lipid transfer proteins (LTPs), β-amylases, ATIs and some gluten proteins can cause immediate-type immunoglobulin E (IgE) mediated allergic reactions^24–29^. Moreover, clinical and functional studies suggest that type 2 food allergies, driven by, e.g., eosinophils and prominently to wheat proteins, play an important role in promoting irritable bowel syndrome^14,30,31^.

Previous studies compared modern and ancient wheat species limited to proteins of a specific family such as gluten proteins^32^ or ATIs^33–35^, or studied the immunogenic potential of ATIs between common wheat and einkorn^21,36^. Compared to earlier gel-based proteomic studies, latest developments in LC-MS-based proteomics allow to quantify thousands of proteins in less than 1.5–2 h per sample^37^. Recently, this proteomic technology was applied to compare the proteomes of few cultivars in common wheat, spelt and rye^38,39^ showing that an important number of proteins were differently expressed even between common wheat and spelt, both being hexaploid species. Comparing the protein expression levels in 150 common wheat cultivars, our recent study demonstrated a large impact of the environment and of different cultivars on the expression of a range of proteins^40^. However, to the best of our knowledge, the flour proteomes of common wheat, spelt, durum, emmer and einkorn have yet not been compared using modern proteomics technology.

In the present work, we utilized high-resolution liquid chromatography-tandem mass spectrometry (LC-MS/MS) based label-free quantitative (LFQ) proteomics to characterize the proteome of the whole-grain flour of ten cultivars for each of five wheat species all grown in three diverse environments. Our objectives were to (i) elaborate a high-resolution reference proteome of five wheat species, (ii) quantify the effects of the species, cultivars within species and the environment on protein abundance / expression level, and (iii) elucidate similarities and differences in the proteomes of different wheat species based on protein patterns related to allergies, immune activation and nutritional quality for improved health and wheat supply chains.

## Results and discussion

In our analysis, we identified 17,277 peptide sequences and 2,896 different proteins across 150 flour samples, representing, to our knowledge, the largest proteome study in cereals to-date. Moreover, the investigation of ten cultivars for each species grown in three diverse environments enabled the in-depth evaluation of the effects of the species, cultivars within species and the environment on the protein expression.

### Basis for future in-depth proteomic research in wheat species

**Fig. 1:**
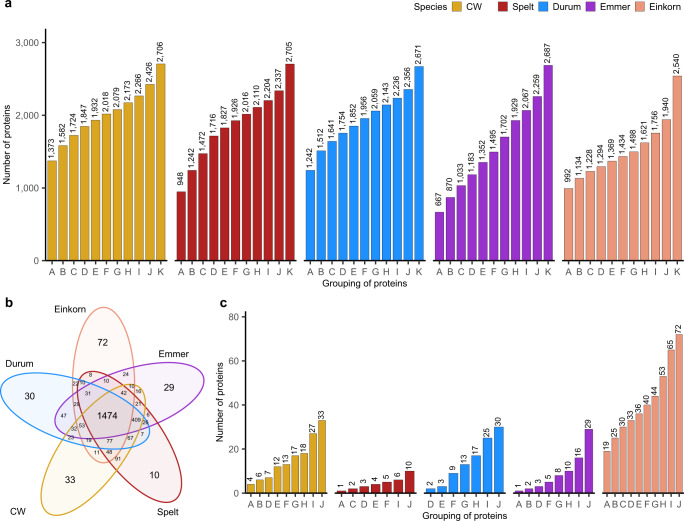
Overview of the identified proteins in five wheat species. **a** Grouping of proteins based on their presence in a certain number of cultivars across three environments. The capital letters on the x-axis denote groups of proteins. These groups correspond to the proteins that were expressed in all of the three environments in all 10 cultivars (A), in at least 9 (B), 8 (C), 7 (D), 6 (E), 5 (F), 4 (G), 3 (H), 2 (I) cultivars and in at least 1 (J) cultivar. Proteins belonging to group K were found in at least one sample. For further analysis, the proteins from group J were considered. **b** Number of proteins that are unique or common between species. **c** Grouping of the unique proteins (from Fig. 1b) based on their presence in a certain number of cultivars across three environments. The letters on x-axis have the same denotation as in Fig. 1a. CW, common wheat.

Our proteomic analyses identified 2706, 2705, 2671, 2687 and 2540 proteins in common wheat, spelt, durum, emmer and einkorn, respectively (Fig. 1a). Interestingly, these numbers were quite similar between species, although the composition of the protein sequence database used for searching the MS spectra was biased towards entries from common wheat (38% of all entries) and durum wheat (51% of all entries) due to the lack of reference proteomes for some of the analyzed species. These findings were in agreement with a study comparing only the hexaploid species common wheat and spelt^38^ indicating a high sequence homology across wheat species irrespective of different ploidy levels.

Overall, protein abundances were highly affected by choice of cultivars within species and the environment, where the cultivars were grown. For instance, from the total number of identified 2,540 proteins in einkorn (Fig. 1a letter K), only 1,940 were stably expressed across all three environments in at least one cultivar (Fig. 1a letter J): Thus, 600 proteins were present/absent only due to environmental effects. Furthermore, for the remaining proteins the mean heritability was 0.24 with only 380 proteins having a heritability higher than 0.5 (Supplementary Fig. 1a). The heritability quantifies the cultivar’s effect on the total expression of a trait and ranges from 0 to 1. The lower the heritability, the higher the impact of the environment vs. the cultivar on a trait’s expression. Consequently, the impact of the environment including soil, climatic factors and cultivation practices on protein expression is very high, which is consistent with the literature^32,34,38,40^. For further discussion, we disregarded proteins, which were only affected by the environment, specifically proteins not stably present across all environments in at least one cultivar of a species (Fig. 1a letter K).

Generally, the different cultivars within a species varied considerably in their protein expression, as evidenced by the presence/absence of proteins (Fig. 1a) or a large coefficient of variation across cultivars for a protein (Supplementary Fig. 1b). For instance, in einkorn 1,940 proteins were identified, which were stably present in at least one cultivar across all three environments (Fig. 1a letter J), but only 992 proteins were present in all 10 cultivars and all environments (Fig. 1a letter A). This is in line with findings of a previous study that compared common wheat and spelt^38^, and highlights the necessity to use representative sets of cultivars within species grown in several environments when measuring protein abundances.

Considering the high environmental impact on protein expression, we elaborated a list of proteins mainly affected by the genetics for future research and breeding. These proteins might be successfully manipulated across future wheat supply chains by choice of cultivars. We therefore selected only proteins within each species, which (i) had a heritability >0.50, (ii) had missing data ≤20%, and (iii) were detected in all environments in ≥50% cultivars and in at least 2 of 3 environments in ≥80% cultivars. These were 845, 611, 863, 262 and 296 proteins in cultivars of common wheat, spelt, durum, emmer and einkorn, respectively (Supplementary Table 1). This list contained proteins from important families such as proteins crucial for baking quality (glutenins, gliadins), the starch pathway (beta-amylases, glucan-branching enzymes, sucrose synthases), confirmed allergens (enzyme inhibitors, serpins, lipid transfer proteins) and the plants’ response to field conditions (heat shock, heat and drought response proteins, late embryogenesis abundant proteins) and others that were partly present across different wheat species (Table 1). Moreover, many proteins on our list of “hot candidate proteins” for future wheat supply chains have still unknown or rather descriptive names warranting urgent future research. Summarizing, our present high-coverage proteomics study provides a solid basis for future in-depth research on proteome and protein functions across different wheat species.

**Table 1:** Potentially interesting proteins for future research and breeding grouped into protein families.

UniProt annotation / keyword	Common wheat	Spelt	Durum	Emmer	Einkorn
Glutenin, HMW, LMW	19 (0.81)	18 (0.78)	15 (0.85)	6 (0.69)	7 (0.78)
Gliadin	23 (0.81)	16 (0.81)	12 (0.81)	2 (0.64)	5 (0.82)
Puroindoline	1 (0.82)	0	0	0	2 (0.54)
Beta-amylase	7 (0.82)	4 (0.93)	2 (0.75)	1 (0.80)	1 (0.62)
1,4-alpha-glucan branching enzyme	3 (0.66)	2 (0.62)	7 (0.74)	2 (0.57)	3 (0.64)
Sucrose synthase, Sucrose_synth	3 (0.78)	3 (0.69)	5 (0.75)	1 (0.65)	0
Alpha-amylase inhibitor, amylase inhibitor	8 (0.71)	7 (0.72)	4 (0.79)	0	0
Trypsin inhibitor	2 (0.83)	3 (0.73)	1 (0.66)	0	1 (0.59)
Serpin	4 (0.75)	4 (0.76)	9 (0.85)	5 (0.60)	1 (0.57)
Non-specific lipid-transfer protein	6 (0.80)	5 (0.89)	4 (0.74)	0	1 (0.77)
Glutathione transferase, S-transferase	2 (0.74)	1 (0.70)	4 (0.80)	2 (0.69)	0
P:defense response^a^	15 (0.74)	11 (0.66)	15 (0.77)	10 (0.71)	8 (0.66)
P:response to water^a^	8 (0.69)	4 (0.60)	4 (0.78)	4 (0.74)	0
Heat shock	5 (0.64)	2 (0.57)	4 (0.73)	5 (0.76)	3 (0.58)
P:response to heat^a^	10 (0.74)	5 (0.65)	17 (0.70)	7 (0.75)	3 (0.63)
Late embryogenesis abundant (LEA)	2 (0.73)	0	0	0	0

*HMW* high molecular weight, *LMW* low molecular weight.

^a^proteins involved in a given biological process (P:) according to the gene ontology (GO) analysis.

These proteins were filtered by applying a stringent criterion within each species, i.e., heritability >0.50, missing data ≤20%, detected in all environments in ≥50% cultivars and in at least 2 of 3 environments in ≥80% cultivars. For each species, the number of proteins within specific protein families fulfilling the filtering criteria are shown with their average heritability in brackets. For more details, see Supplementary Table1.

### Five wheat species can be separated by their patterns of protein expression

**Fig. 2:**
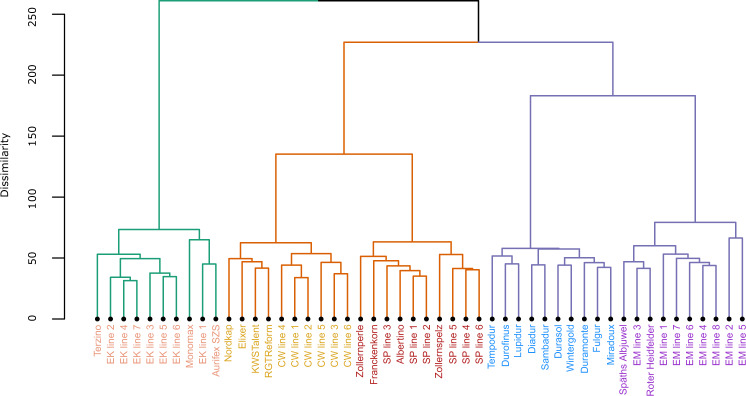
Hierarchical clustering of 50 cultivars belonging to five wheat species. The colors of the lines (branches) highlight three clusters of cultivars corresponding to their ploidy level. The colors of the cultivar labels correspond to the color legend for species in Fig. 1a. In total, 2774 proteins were used for clustering, which included proteins unique to one of the five species and proteins common between species. All those proteins were identified across all three environments in at least one cultivar and hence belonged to the group J in Fig. 1a.

The hierarchical clustering of the 50 cultivars from five wheat species using 2,774 proteins clearly separated the cultivars into five groups corresponding to the five species (Fig. 2). The clustering reflected the genetic distance between the species by depicting smaller distances between species with the same ploidy level. For instance, the two hexaploid species common wheat and spelt clustered more closely together than einkorn and common wheat, as did the two tetraploid species durum and emmer, which underlines the validity of our proteomic workflow.

**Fig. 3:**
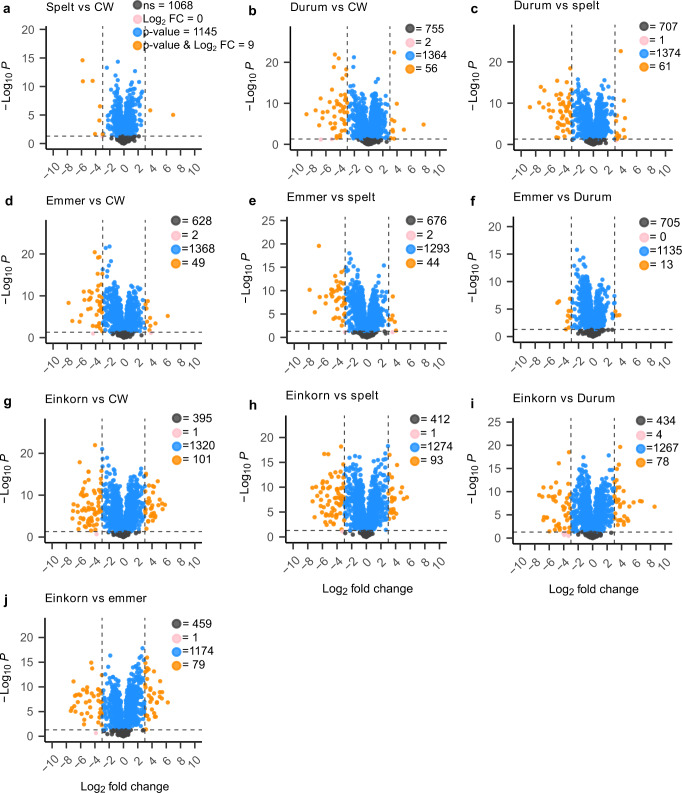
Results of the comparisons for individual proteins between different pairs of wheat species. **a–j** Volcano plots of proteins that were common between different pairs of wheat species. The x-axis shows the log2-scale fold change (Log2FC) in the LFQ abundance of proteins between two species. The y-axis shows the negative log10-scale *p* value from the t-test. The vertical dashed lines denote ±3 Log2FC threshold, while the horizontal dashed line denotes *p* value threshold of 0.05, e.g., the orange points in (**a**) show proteins, which are downregulated (Log2FC < -3) or upregulated (Log2FC > 3) in spelt compared to common wheat and passed both thresholds i.e., *p* value and Log2FC. Color of the points represents significance status of proteins for *p* value (<0.05) and Log2FC (±3): gray, non-significant for both thresholds; pink, significant Log2FC only; blue, significant *p* value only; orange, significant for both thresholds. CW, common wheat.

This separation was further corroborated by proteins unique to an individual species or present only in few but not all species (Fig. 1b, c; Supplementary Fig. 2). For instance, the diploid einkorn had the highest number of unique proteins with ≥40 unique proteins present in at least four einkorn cultivars. By contrast, 1,474 proteins were jointly expressed across all five wheat species (Fig. 1b), with the highest (lowest) number of proteins expressed jointly in pairwise comparisons between common wheat vs. spelt (einkorn vs. emmer; Supplementary Fig. 2). However, >50% of the joint proteins between any pair of species were expressed with a statistically significantly different abundance (Fig. 3), showing a tendency that the larger the difference between the ploidy levels of the species, the higher the percentage of differentially expressed proteins. For instance, 52% of the joint proteins between spelt and common wheat (Fig. 3a) showed a significantly different expression, which reached 78% for einkorn vs. common wheat (Fig. 3g). Thereby a higher number of proteins was downregulated than upregulated in einkorn compared to the other wheat species (Supplementary Fig. 3).

Overall, 254 proteins showed not only a statistically different expression between different pairs of species but also passed a stringent threshold of ±3 log_2_ fold change (equivalent to an 8-fold up/downregulation) (Fig. 3 orange points, Supplementary Table 2). These proteins belonged to the important protein families mentioned above (baking quality, starch pathway, allergens, the plants’ stress response) (Table 2). Except for our recent comparison between common wheat and spelt^38^, no such analysis has been published to-date.

**Table 2:** Proteins with potentially important functions, which were significantly differently expressed (t-test, *p* value <0.05) between species.

UniProt annotation / keyword	NPS	GO annotations^a^	NPU				
			Cw	Sp	Du	Em	Ei
Glutenin, HMW, LMW	50	F:nutrient reservoir activity	2	–	1	1	3
Gliadin	41	F:nutrient reservoir activity	6	2	2	–	4
Puroindoline	7	F:nutrient reservoir activity	–	–	–	–	–
Beta-amylase	7	F:beta-amylase activity	1	–	–	1	–
1,4-alpha-glucan branching enzyme	12	P:glycogen biosynthetic process	1	–	1	–	–
Starch synthase	12	11 F:glycogen (starch) synthase activity	–	–	1	–	1
Sucrose synthase, Sucrose_synth	8	F:sucrose synthase activity	–	–	–	–	–
Alpha-amylase inhibitor, amylase inhibitor, Trypsin inhibitor, BBTTI, CTI	30	F:serine-type endopeptidase inhibitor activity	1	–	–	–	1
Serpin	16	F:serine-type endopeptidase inhibitor activity	1	–	–	–	–
Nonspecific lipid-transfer protein	10	P:lipid transport	–	–	–	–	–
ATP synthase	14	P:ATP synthesis coupled proton transport	–	–	–	–	–
Glutathione transferase, S-transferase	8	7 P:glutathione metabolic process, 1 F:glutathione dehydrogenase (ascorbate) activity	–	–	–	–	–
Defensin, Barwin, Thaumatin-like, Thionin, Knot1	13	P:defense response	–	–	1	–	2
Dehydrin	4	P:response to water	–	1	–	–	–
Heat shock, SHSP	42	F:ATP hydrolysis activity, P:response to heat	4	–	7	3	6
Late embryogenesis abundant, LEA	4	-	–	–	–	–	–

*NPS* number of proteins, which were significantly differently expressed (t-test, *p* value <0.05) between species, *GO* gene ontology, *NPU* number of proteins, which were unique to only one of the five species, *Cw* common wheat, *Sp* spelt, *Du* durum, *Em* emmer, *Ei* einkorn, *HMW* high molecular weight, *LMW* low molecular weight, *BBTTI* Bowman-Birk type trypsin inhibitor, *CTI* chymotrypsin inhibitor.

For more details, see Supplementary Table 2.

^a^GO annotations provided as either molecular function (F:) or biological process (P:) obtained from the Gene Ontology (GO) analysis.

Owing to the lack of reference proteomes for spelt, emmer and einkorn, our study might be biased in the way that proteins unique to these species have not been detected, which may further increase the differences among species. However, to date our study provides the highest proteome coverage regarding the identified and quantified proteins across five wheat species.

### Potential and known allergenic proteins are largely reduced in einkorn

Wheat is an important and usually healthy staple crop for human and animal nutrition, but a sizable population suffers from inflammatory wheat sensitivities. These are celiac disease, IgE-mediated and non-IgE-mediated (type 2) wheat allergy, and innate immune activation by ATI-proteins, the latter two possibly contributing to non-celiac wheat sensitivity (NCWS)^14,17–23,25,30,31,41–45^. As most potential allergens are proteins and large differences in the proteomes of the different wheat species were elaborated above, we investigated the distribution of potential allergenic proteins across the wheat species in more detail. We followed the approach of Zimmermann et al.^39^ to compile a list of allergens based on the information from data about seed-borne wheat allergens^27^ and the allergome database (http://www.allergome.org/index.php)^46^, and additionally included ATIs.

**Fig. 4:**
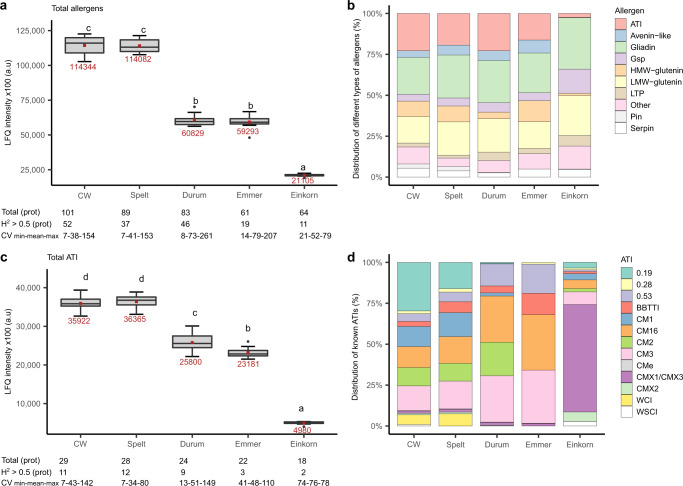
Allergenic proteins in wheat species. LFQ intensity/abundance of different families of proteins per cultivar in different wheat species for (**a**) potential allergens, and (**c**) proteins identified as ATIs. Boxes with different letters indicate significant differences between the wheat species (ANOVA with Tukey’s test, *p* < 0.05). Each box summarizes the content of 30 samples (ten cultivars grown at three locations). Mean values for each species are highlighted in red color. Furthermore, within each species the distribution of (**b**) allergens, and (**d**) ATIs with a known type is shown. CW, common wheat; Total (prot), number of allergenic proteins or ATIs used to calculate total LFQ intensity (%); H^2^ > 0.5 (prot), number of allergenic proteins or ATIs with heritability >0.5; H^2^, heritability; CV_min-mean-max_, minimum-mean-maximum values of the coefficient of variation (%) for allergenic proteins or ATIs with heritability >0.5; LFQ, label-free quantification; ATI, amylase/trypsin inhibitor; Gsp, grain softness protein; HMW, high molecular weight; LMW, low molecular weight; LTP, lipid transfer protein; Pin, puroindoline; BBTTI, Bowman-Birk type trypsin inhibitor; CM, chloroform/methanol soluble protein; WCI, wheat chymotrypsin inhibitor; WSCI, wheat subtilisin-chymotrypsin inhibitor.

The sum of all these potential allergenic proteins was clearly different among the species and corresponded almost perfectly with the ploidy levels (Fig. 4a). While their total abundance was similar in hexaploid common wheat and spelt, they were roughly reduced two-fold in tetraploid durum and emmer, and 5.4-fold in diploid einkorn. These differences were due to both the different numbers of potential allergens and different protein abundances. By contrast, total grain protein content (GPC) was slightly higher in einkorn, spelt and emmer compared to common wheat (Supplementary Fig. 4), which is in line with prior findings^32,47^, with differences in GPC of other species compared to common wheat ranging between 1% and 20%. In common wheat, spelt and durum, almost half of the potential allergenic proteins had a heritability >0.5, and across all species their coefficient of variation with heritability >0.5 ranged between 7% and 261% (Fig. 4a). Therefore, the abundance of these potential allergens can be reduced in a targeted way using proteomics to monitor breeding and cultivar choice, which confirms recent findings on gluten and ATI composition across different wheat cultivars^33,34,48^. This would, however, require the development of rapid test methods, which can be used in daily business across wheat supply chains. Our reference proteome can be used as starting point, e.g., by concentrating on allergens with high heritability and coefficient of variation across cultivars within a species.

Besides the quantity of allergens, their distribution within species also varied considerably (Fig. 4b). While the allergens of common wheat were largely represented by ATIs, gliadins, HMW and LMW glutenins, more than 50% of the einkorn allergens (substantially lower abundances than in other wheat species, Fig. 4a) were comprised of gliadins and LMW glutenins. Consistent with the published literature^33,48^, einkorn had significantly lower quantity of ATIs compared to the other wheat species i.e., 7.2, 7.3, 5.2 and 4.7-fold lower than in common wheat, spelt, durum and emmer, respectively (Fig. 4c). The ATIs are implicated as major allergens and also as activators of TLR4 in animal models of diseases^17–19,21–23,33,48^. Einkorn ATIs were mainly attributable to CMX1/CMX3 (UniProt accession M8A1S2) (Fig. 4d). Interestingly, in einkorn the predominant ATIs of type CMX1/CMX2/CMX3 have not been described to inhibit amylase activity^49^ and CMX1/CMX3 are neither listed as seed-borne wheat allergens^27^ nor in the allergome database (http://www.allergome.org/index.php)^46^. Furthermore, another ATI (UniProt accession C5J3R4; Description, Trypsin inhibitor OS = Triticum monococcum subsp. monococcum OX = 408188 GN = Eti-Am1) was found only in emmer and einkorn, confirming our recent analyses focused on ATIs^35^. By contrast, ATIs of hexaploid common wheat and spelt, were mainly represented by 0.19, CM1, CM2, CM3 and CM16, whereas in tetraploid durum and emmer the ATIs were mainly 0.53, CM3 and CM16 (Fig. 4d). Iacomino et al^36^ showed that the einkorn ATIs were more susceptible to in vitro enzymatic hydrolysis than the fairly pepsin-trypsin resistant ATIs of tetra- and hexaploid wheats^19,21–23,50^, and therefore largely proteolytically degraded during food processing and especially upper gastrointestinal passage, resulting in absent or reduced ability to trigger innate immunity^36^. Similarly, Sievers et al.^51^ showed that einkorn could be beneficial for people who are sensitive only to ATIs but might not be safe for individuals suffering from wheat allergy.

Summarizing, our study demonstrates a much lower abundance of potential allergens and ATIs in einkorn, and also in durum and emmer in comparison with hexaploid common wheat and spelt. However, allergen databases appear to be biased towards having more allergens from common wheat than from other wheat species probably due to the limited use and cultivation of alternative wheat species such as spelt, emmer and einkorn. Furthermore, the utilized proteome reference included only the reference proteomes of *T. turgidum* ssp. *durum* (51% of all database entries), *T. aestivum* ssp. *aestivum* (38% of all database entries) and *T. urartu* (9% of all database entries). Consequently, our approach might have fallen short to identify all potential allergens from einkorn, emmer, durum and spelt. Nevertheless, we speculate that emmer, durum and einkorn still have a considerably lower number and lower abundance of allergenic proteins, because the identified differences are very large and sequence homology was high so that high number of proteins could be identified in all wheat species regardless of the limitations mentioned above.

The need to find alternative cereal crops with reduced allergenicity or ATIs is highlighted by the increasing number of preclinical and especially clinical studies comparing different wheats. First explorative preclinical studies suggest a potential better tolerability of ancient vs. modern wheat cultivars for patients with wheat allergy and NCWS. In these studies, ancient diploid and also tetraploid wheats like einkorn and emmer were indeed better tolerated by NCWS patients, many of whom had either an IgE positive or an IgE negative (type 2) wheat allergy, when compared to modern hexaploid wheats^18,52–58^. Similarly, Picascia et al.^59^ investigated the effect of the diet prepared from einkorn and common wheat flours on the immune response in celiac disease patients and concluded that einkorn caused a lower in vivo T-cell response in comparison with common wheat. However, these studies were not based on representative samples including different cultivars from different wheat species grown at comparable environmental conditions, all of them largely influencing the proteomic profiles of flour samples as indicated in this study. Consequently, better targeted controlled clinical trials using wheats with a defined low content of potential allergens are urgently needed and can be started based on the reference proteomes delivered by current study. Besides einkorn and other wheat cultivars with low abundance of potential allergens, the effect of different flour processing and bread making procedures, such as long sourdough fermentation, on the abundance and activity of allergens should also be investigated in pursuit of identifying healthier wheat products especially for people with wheat related disorders.

### Outlook: Einkorn as sustainable crop for marginal environments, but potential health benefits have to be urgently validated

In addition to the lower amount of potential allergens with yet lacking clinical proof described above, einkorn contains more protein and considerably higher amounts of valuable trace compounds compared with common wheat, such as vitamin E, luteins, steryl ferulates^60,61^, minerals like Fe and Zn^62^ in addition to several other minerals such as Ca, Cu, K, Mg, Mn, P, and S^63^ – all these compounds being important for a healthy diet^61,62^. However, the bioavailability of these compounds in breads and other cereal products and their impact on human health have yet not been investigated warranting urgent further research.

Agronomically, einkorn shows a considerably higher protein yield efficiency than common wheat^32^, almost complete resistance against fungi^8^ and flexibility to sow the same cultivar before or after winter, which does not exist in other cereals. However, compared to common wheat, einkorn plants are taller and, thus, more prone to lodging than common wheat. Furthermore, einkorn has almost 70% less grain yield under good soil conditions than common wheat^47^. Owing to the necessity of increasing agricultural productivity per available cultivated land to feed an increasing world population, einkorn cannot replace widely cultivated common wheat. Nevertheless, in marginal environments the productivity of common wheat is markedly reduced^64^, whereas einkorn performs well^65^. The marginal environments include sandy soils and higher altitudes in mountainous regions and/or lack of nitrogen fertilizer due to high costs, environmental restrictions or organic farming. Consequently, considering the lowest allergen and ATI contents and the high amounts of nutritious ingredients of einkorn, clinical trials are urgently needed to validate these potential health benefits, as einkorn could be a promising sustainable alternative crop for marginal regions enhancing agro-biodiversity.

## Methods

### Plant material and field trials

We investigated 10 cultivars of each of the five species of wheat namely common wheat (*T. aestivum* ssp. *aestivum*, 2n = 6× = 42, A^u^A^u^BBDD), spelt (*T. aestivum* ssp. *spelta*, 2n = 6× = 42, A^u^A^u^BBDD), durum (*T. turgidum* ssp. *durum*, 2n = 4× = 28, A^u^A^u^BB), emmer (*T. turgidum* ssp. *dicoccum*, 2n = 4× = 28, A^u^A^u^BB), and einkorn (*T. monococcum* ssp. *monococcum*, 2n = 2× = 14, A^m^A^m^). The selected cultivars of each wheat species include very important cultivars representing the recent market in Germany. In addition, the best and latest breeding lines available from multiple environment field trials were added to the selected cultivars (Supplementary Table 3).

The field trials were conducted as winter cropping, i.e., sowing in October 2018 and harvest in July 2019, at three diverse locations for each species in Germany/Austria. The trial locations with their GPS coordinates for each species are provided within brackets followed by name of the species, such as, common wheat (DSV-Leutewitz - 51°8'58’N, 13°21'46.908“E; Eckartsweier - 48°31'45“N, 7°51'18“E; Stuttgart-Hohenheim - 48°42'50“N, 9°12'58“E), spelt (Eckartsweier - 48°31'45“N, 7°51'18“E; Stuttgart-Hohenheim - 48°42'50“N, 9°12'58“E; Oberer Lindenhof - 48°28'26“N, 9°18'12“E), durum (Eckartsweier - 48°31'45“N, 7°51'18“E; Stuttgart-Hohenheim - 48°42'50“N, 9°12'58“E; Probstdorf - 48°10'19“N, 16°37'13“E), emmer (Stuttgart-Hohenheim - 48°42'50“N, 9°12'58“E; Ihingerhof - 48°44'44“N, 8°55'23“E; Oberer Lindenhof - 48°28'26“N, 9°18'12“E), einkorn (Eckartsweier - 48°31'45“N, 7°51'18“E; Ihingerhof - 48°44'44“N, 8°55'23“E; Oberer Lindenhof - 48°28'26“N, 9°18'12“E). The wheat species were investigated in separate but adjacent trials at individual locations using an un-replicated field design randomized separately for each species across test locations. All trials received the same field treatments of intensive conventional farming practices except for nitrogen (N) fertilization, where common wheat, durum, spelt, emmer and einkorn were fertilized to reach the following level of N including N measured in the soil (“Nmin”): 180, 180, 160, 60 and 60 kg N/ha, respectively. This individual adjustment was done to reflect recent agricultural practice in conventional production. Furthermore, owing to its field resistance to fungal diseases einkorn did not receive any fungicide treatment in contrast to all other species. Field net plot size was 5 m² in all locations. All plots were machine-sown and combine-harvested. All samples of spelt, emmer and einkorn were dehulled and cleaned using a Mini-Petkus seed cleaner (Röber, Bad Oeynhausen, Germany) to separate hulls, straw and damaged kernels. Dehulling was performed using a classical stone mill, in which the stone was replaced by hard rubber. For common wheat, seed cleaning was also performed using the Mini-Petkus seed cleaner in order to remove chaff and straw particles, which were still present after combine harvesting.

### Laboratory analyses

Three observations (samples) from three diverse locations were used to calculate the mean abundance of a protein per cultivar of each species. However, for lab analysis we used one technical replicate for each sample. While the use of more technical replicates is preferable, we had to compromise due to the large number of samples to analyze under a given budget and time-limit. This approach is justified, since from numerous studies on different traits in field trials, it is well known to and accepted by the scientific community that a large variance in data arises due to differences in the conditions between different growing locations. Given this location-dependent variability, the data quality improves and becomes more representative for general statements about the expression of a trait, such as proteins in different species, if the number of locations is increased at the expense of the number of technical replicates and not vice versa. Furthermore, the low number of technical replicates was accounted for during the statistical analysis to estimate the mean values across three locations by adjusting for field trial and effects of lab analyses.

#### Protein extraction

From each cultivar of common wheat, spelt, durum, emmer and einkorn 20 mg of whole-grain flour were weighed into 1.5 mL plastic tubes (Protein LoBind tubes, Eppendorf, Hamburg, Germany). Next, 50 µL of LC-MS grade water were added and the tubes were vortexed until the flour was completely resuspended. Immediately afterwards, 950 µL of extraction buffer composed of 7 M urea, 2 M thiourea, 2% (w/v) CHAPS, 5 mM dithiothreitol (DTT) and LC-MS grade water were added and the tubes were vortexed again. After incubation at 22 °C for 10 min, the samples were centrifuged at 16,000 x *g* and 22 °C for 10 min. The clear upper layer of the supernatants was used for all subsequent steps. Average protein concentrations were determined from extracts of species-specific flour mixtures using the Pierce 660 nm protein assay (Thermo Scientific, Rockford, IL, USA; Supplementary Fig. 4).

#### Sample preparation

Proteins were purified and digested into LC-MS-compatible tryptic peptides using a filter-assisted sample preparation protocol (FASP)^66,67^ as described before^68^ with minor modifications. Briefly, 30 µg of protein extracts were loaded onto centrifugal ultrafiltration devices (Nanosep with 30 K MWCO Omega membrane 30 K MWCO, Pall, Port Washington, NY, USA) and centrifuged at 16,000 x *g* and 22 °C for 15–30 min until the liquid completely passed through the membrane. Disulfide bonds were reduced using DTT followed by alkylation of free cysteines using iodoacetamide (IAA). The alkylation reaction was quenched by the addition of DTT. After each step, the membrane was washed once using a buffer containing 8 M urea and 100 mM Tris-HCl (pH 8.5). Finally, buffer exchange was performed washing the membrane three times with a buffer containing 50 mM ammonium bicarbonate and LC-MS grade water. On-filter tryptic digestion was performed by the addition of trypsin (Trypsin Gold, Promega, Madison, WI, USA) at a protease-to-protein ratio of 1:50 (w/w) and overnight incubation at 37 °C. Tryptic peptides were collected into fresh tubes by centrifugation and an additional wash of the membrane using 50 mM ammonium bicarbonate. The flow through was acidified adding trifluoroacetic acid (TFA) to achieve final concentration of 0.5% (v/v). Peptides were loaded onto Sep-Pak tC18 96-well cartridges (Waters Corporation, Milford, MA, USA) and desalted using 0.1% (v/v) TFA in LC-MS grade water as wash solvent and 0.1% (v/v) TFA in 50% (v/v) acetonitrile/water as elution solvent. Purified peptides were lyophilized and reconstituted in 20 µL of 0.1% (v/v) formic acid (FA) in LC-MS grade water prior to LC-MS analysis.

#### Liquid chromatography-mass spectrometry

Tryptic peptides of each sample were sequentially analyzed by liquid chromatography-tandem mass spectrometry (LC-MS/MS) using a nanoACQUITY UPLC system (Waters Corporation) coupled to a SYNAPT G2-S mass spectrometer (Waters Corporation) via a NanoLockSpray dual electrospray ionization source (Waters Corporation). Microflow LC and source interface were set up as described before^69^. Peptides in amounts of 1.5 µL were loaded onto an HSS-T3 C18 reversed phase column (Waters Corporation) with a length of 250 mm and an inner diameter of 300 µm and separated by gradient elution using LC flow rates of 5 µL/min and 60 min LC methods. Precursor and fragment ion mass spectra were recorded using an ion mobility-enhanced data-independent acquisition strategy as described before (UDMS^E^)^68^.

#### Data processing and label-free quantification

Raw UDMS^E^ data were processed using ProteinLynx Global Server v3.0.2 (PLGS, Waters Corporation) and searched against a database compiled of proteins of the genus *Triticum* (UniProtKB release 2020_05, taxon ID: 4564, 367,831 entries) including the reference proteomes of *T. turgidum* ssp. *durum* (51% of all database entries), *T. aestivum* ssp. *aestivum* (38% of all database entries) and *T. urartu* (9% of all database entries) plus 171 common MS contaminants using following parameters: Trypsin was specified as digestion enzyme, two missed cleavages per peptide were allowed for initial database search, carbamidomethylation of cysteines was set as fixed, and methionine oxidation as variable modification. The false discovery rate (FDR) was calculated in PLGS by searching a database of reversed protein sequences and a cutoff of 0.01 was applied.

Label-free quantification (LFQ) including retention time alignment, feature clustering, cross-run normalization and protein inference was performed using ISOQuant v1.8^68^. Only peptides without missed cleavages, a minimum sequence length of seven amino acids, a minimum PLGS score of 6.0 and no variable modification were considered for quantification. An FDR cutoff of 0.01 was applied at the peptide and protein level in ISOQuant, ensuring a 1% FDR on dataset level. Proteins identified by at least two different peptides were quantified by averaging the intensities of the three peptides with the highest intensities belonging to the respective protein (Top3 method)^70^. Abundances of shared peptides were redistributed between proteins based on relative abundances of uniquely assigned peptides (see ISOQuant manual for details; http://www.immunologie.uni-mainz.de/isoquant/index.php?slab=user-manual#x1-400006.7). TOP3-based quantification provides an estimate of the total amount of each protein in a sample. By summing up over all detected and quantified proteins, the relative amount of each protein in the respective proteome (i.e. parts per million of total protein) can be determined. This value is independent of the total amount of protein in the sample or on column.

#### Database search and homology filtering - detailed procedure

During PLGS database search, each detected peptide is mapped to all proteins in the database containing the respective peptide. This is initially performed on a run-by-run basis in PLGS. Subsequently, during the data processing in ISOQuant, protein groups are filtered on entire dataset basis (i.e. taking peptide and protein information from all runs, filtering at 1% FDR on peptide level initially and taking only peptides without missed cleavages, a minimum sequence length of seven amino acids, a minimum PLGS score of 6.0 and no variable modification), then based on the Occam’s Razor principle, resulting in a reduced protein list (also filtered at 1% FDR) that can explain all peptides passing the above criteria in the dataset.

#### Blast2GO analysis

Automatic functional annotation of proteins identified by LC-MS/MS was performed using the software tool Blast2GO v5.2.5^71^. Blast2GO uses the BLAST algorithm to identify similar proteins and transfers already existing Gene Ontology (GO) annotations to the queried protein sequences. In addition, InterProScan is used to obtain protein family and domain information which are converted and merged to GOs. Blast searches were performed against the NCBI database of non-redundant protein sequences (nr) using the blastp algorithm. Otherwise, the Blast2GO workflow (Blast, InterProScan, mapping and annotation) was carried out with standard parameters.

### Phenotypic data analysis

Phenotypic data analysis was performed separately for each species according to the linear mixed model, given in Eq. (1):

1 $${{{\boldsymbol{y}}}}_{{{{\boldsymbol{ik}}}}} = {{{\boldsymbol{u}}}} + {{{\boldsymbol{v}}}}_{{{\boldsymbol{i}}}} + {{{\boldsymbol{env}}}}_{{{\boldsymbol{k}}}} + {{{\boldsymbol{e}}}}_{{{{\boldsymbol{ik}}}}},$$

where *y*_ik_ is the phenotypic (= measured) observation for the *i*th cultivar tested in the *k*th environment, *u* is the general mean, *v*_i_ the effect of the *i*th cultivar, *env*_k_ the effect of the *k*th environment, and *e*_ik_ is the residual error. Variance components, which are variances due to cultivars, environments and residual error, were estimated using the restricted maximum likelihood (REML) method assuming a random model in a classical one-stage analysis^72^. A likelihood ratio test with model comparisons was performed^73^ to check for significance of the variance components. Average values of the proteins across the different environments were determined as best linear unbiased estimates (BLUEs) assuming fixed genetic (cultivar) effects. Heritability estimates (*h*^2^) were computed following Piepho and Möhring^74^ as given in Eq. (2):

2 $${{{\boldsymbol{h}}}}^2 = 1 - \frac{\vartheta }{{2\sigma _{{{\boldsymbol{G}}}}^2}},$$

where ϑ is the mean variance of a difference of two best linear unbiased predictors and $\sigma _G^2$ the genotypic variance (cultivar variance). All analyses were performed utilizing the statistical software R^75^ and ASReml 3.0^76^.

### Comparison of the proteomes of different wheat species

For the t-test, BLUEs of proteins per cultivar in each species were used. We implemented Student’s t-test (α = 0.05)^77^ to compare the abundance of proteins between each pair of wheat species. For the t-test, assumption of the equality of variances between groups was examined by applying Levene’s test^78^. If Levene’s test was significant (*p* < 0.05, meaning that the variances between groups were not equal), then the more robust Welch’s t-test was conducted instead of the regular t-test, with corrected degrees of freedom. Student’s t-test and Levene’s test were implemented using the statistical software R^75^.

Volcano plots were generated for each pair of species to identify proteins, which abundance was statistically significantly different (t-test, p < 0.05) and was above/below an arbitrary threshold of ±3 log_2_ fold change (Log_2_FC). R-package *EnhancedVolcano* was used to produce volcano plots. Log_2_FC was calculated using the formula given in Eq. (3):

3 $${{{\mathbf{Log}}}}_2{{{\mathbf{FC}}}}_{{{\boldsymbol{p}}}} = {{{\boldsymbol{u}}}}_{{{\boldsymbol{i}}}} - {{{\boldsymbol{u}}}}_{{{\boldsymbol{j}}}},$$

where Log_2_FC_*p*_ is the abundance of protein *p* in species *i* relative to species *j*, *u*_*i*_ and *u*_*j*_ are the log_2_ mean abundance of protein *p* in species *i* and species *j*, respectively.

For hierarchical clustering the data were scaled and Euclidian distance was calculated. Hierarchical clustering was performed using “hclust” function in statistical software R^75^ by implementing Ward’s method^79^.

### Identification of allergenic proteins

We used the list of seed-borne wheat allergens^27^ curated based on the databases of allergenic protein families, AllFam (www.meduniwien.ac.at/allfam) and AllergenOnline (www.allergenonline.org) to identify potential allergenic proteins in flour samples from wheat species in the current study. We further extended the identification of allergenic proteins using the comprehensive allergome database (http://www.allergome.org/index.php)^46^, which contains identified, characterized and peer-reviewed allergenic proteins including proteins from the official Allergen Nomenclature of World Health Organization (WHO) and International Union of Immunological Societies (IUIS) (http://www.allergen.org/index.php). The UniProt accessions were used to map proteins to the allergome database through the frontend browser of the UniProt database (https://www.uniprot.org/) and the corresponding Allergome IDs were retrieved (Supplementary Table 4). In addition to the identification of allergenic proteins using the aforementioned databases, we used the protein annotations from the UniProt database to search for amylase/trypsin inhibitors (ATIs) among proteins quantified in this study. The list of ATIs is provided in Supplementary Table 4.

### Reporting summary

Further information on research design is available in the Nature Research Reporting Summary linked to this article.

## Supplementary information

Reporting Summary

Supplementary Information

Supplementary Table 1

Supplementary Table 2

Supplementary Table 3

Supplementary Table 4

## Data Availability

The mass spectrometry proteomics data, including raw files, peptide and protein quantification reports, have been deposited to the ProteomeXchange Consortium via the PRIDE partner repository^80^ with the dataset identifier PXD028676.
