# Supplementary material for: Reference proteomes of five wheat species as starting point for future design of cultivars with lower allergenic potential
Source: NPJ Sci Food. 2023 Mar 25;7:9. doi: 10.1038/s41538-023-00188-0 (PMC10039927; doi:10.1038/s41538-023-00188-0)
Supplement: Supplementary file 1 — Reporting Summary [file 41538_2023_188_MOESM1_ESM.pdf]

nature portfolio

Corresponding author(s): C. Friedrich H. Longin

Last updated by author(s): Feb 16, 2023

Reporting Summary

Nature Portfolio wishes to improve the reproducibility of the work that we publish. This form provides structure for consistency and transparency in reporting. For further information on Nature Portfolio policies, see our Editorial Policies and the Editorial Policy Checklist.

Statistics

For all statistical analyses, confirm that the following items are present in the figure legend, table legend, main text, or Methods section.

n/a	Confirmed	
☐	☒	The exact sample size (*n*) for each experimental group/condition, given as a discrete number and unit of measurement
☐	☒	A statement on whether measurements were taken from distinct samples or whether the same sample was measured repeatedly
☐	☒	The statistical test(s) used AND whether they are one- or two-sided<br>*Only common tests should be described solely by name; describe more complex techniques in the Methods section.*
☐	☒	A description of all covariates tested
☐	☒	A description of any assumptions or corrections, such as tests of normality and adjustment for multiple comparisons
☐	☒	A full description of the statistical parameters including central tendency (e.g. means) or other basic estimates (e.g. regression coefficient) AND variation (e.g. standard deviation) or associated estimates of uncertainty (e.g. confidence intervals)
☐	☒	For null hypothesis testing, the test statistic (e.g. *F*, *t*, *r*) with confidence intervals, effect sizes, degrees of freedom and *P* value noted<br>*Give P values as exact values whenever suitable.*
☒	☐	For Bayesian analysis, information on the choice of priors and Markov chain Monte Carlo settings
☒	☐	For hierarchical and complex designs, identification of the appropriate level for tests and full reporting of outcomes
☒	☐	Estimates of effect sizes (e.g. Cohen's *d*, Pearson's *r*), indicating how they were calculated

Our web collection on statistics for biologists contains articles on many of the points above.

Software and code

Policy information about availability of computer code

Data collection	Data was collected using Excel and other standard software for LC-MS/MS experiments as describe in the Methods section.
Data analysis	Automatic functional annotation of proteins identified by LC-MS/MS was performed using the software tool Blast2GO v5.2.5. All analyses were performed utilizing the statistical software R (v3.3.2) and ASReml-R (v3)an r-package for the standalone software ASReml. The t test and Levene's test were implemented using he statistical software R (v3.3.2).

For manuscripts utilizing custom algorithms or software that are central to the research but not yet described in published literature, software must be made available to editors and reviewers. We strongly encourage code deposition in a community repository (e.g. GitHub). See the Nature Portfolio guidelines for submitting code & software for further information.

Data

Policy information about availability of data

All manuscripts must include a data availability statement. This statement should provide the following information, where applicable:

- Accession codes, unique identifiers, or web links for publicly available datasets
- A description of any restrictions on data availability
- For clinical datasets or third party data, please ensure that the statement adheres to our policy

The mass spectrometry proteomics data, including raw files, peptide and protein quantification reports, have been deposited to the ProteomeXchange Consortium via the PRIDE partner repository with the dataset identifier PXD028676.

Human research participants

Policy information about studies involving human research participants and Sex and Gender in Research.

Reporting on sex and gender	Not applicable.
Population characteristics	Not applicable.
Recruitment	Not applicable.
Ethics oversight	Not applicable.

Note that full information on the approval of the study protocol must also be provided in the manuscript.

Field-specific reporting

Please select the one below that is the best fit for your research. If you are not sure, read the appropriate sections before making your selection.

☒ Life sciences ☐ Behavioural & social sciences ☐ Ecological, evolutionary & environmental sciences

For a reference copy of the document with all sections, see nature.com/documents/nr-reporting-summary-flat.pdf

Life sciences study design

All studies must disclose on these points even when the disclosure is negative.

Sample size	We investigated 10 cultivars of each of the five species of wheat namely common wheat, spelt, durum, emmer, and einkorn. Each of the cultivar was grown at three diverse locations, thus making sample size of 30 for each species. In total there were 150 samples. For large-scale modern proteomics, it is challenging to handle huge number of samples. Simultaneously, increasing the sample size results in steep financial costs, which are not always possible to pay for given that the funding is limited. To the best of our knowledge, previous proteomics studies contained even way fewer samples compared with our study. Keeping in view the aforementioned factors, the sample size in our study can be considered appropriately large.
Data exclusions	Six of the 150 samples were lost during preparing grains for flour production. Therefore, the final data contained 144 of 150 samples. This exclusion was not preplanned.
Replication	Three observations (samples) from three diverse locations were used to calculate the mean abundance of a protein per cultivar of each species. However, for lab analysis we used one technical replicate for each sample. While the use of more technical replicates is preferable, we had to compromise due to the large number of samples to analyze under a given budget and time-limit. This approach is justified, since from numerous studies on different traits in field trials, it is well known to and accepted by the scientific community that a large variance in data arises due to differences in the conditions between different growing locations. Given this location-dependent variability, the data quality improves and becomes more representative for general statements about the expression of a trait, such as proteins in different species, if the number of locations is increased at the expense of the number of technical replicates and not vice versa. Furthermore, the low number of technical replicates was accounted for during the statistical analysis to estimate the mean values across three locations by adjusting for field trial and effects of lab analyses.
Randomization	The wheat species were investigated in separate but adjacent trials at individual locations using an un-replicated field design randomized separately for each species across test locations.
Blinding	Blinding was not relevant to our study because we investigated plant material in randomized field experiments followed by LC-MS/MS. No animals or humans were used as study subjects.

Reporting for specific materials, systems and methods

We require information from authors about some types of materials, experimental systems and methods used in many studies. Here, indicate whether each material, system or method listed is relevant to your study. If you are not sure if a list item applies to your research, read the appropriate section before selecting a response.

Materials & experimental systems

n/a	Involved in the study
☒	☐ Antibodies
☒	☐ Eukaryotic cell lines
☒	☐ Palaeontology and archaeology
☒	☐ Animals and other organisms
☒	☐ Clinical data
☒	☐ Dual use research of concern

Methods

n/a	Involved in the study
☒	☐ ChIP-seq
☒	☐ Flow cytometry
☒	☐ MRI-based neuroimaging
