# Supplementary material for: Reference proteomes of five wheat species as starting point for future design of cultivars with lower allergenic potential
Source: NPJ Sci Food. 2023 Mar 25;7:9. doi: 10.1038/s41538-023-00188-0 (PMC10039927; doi:10.1038/s41538-023-00188-0)
Supplement: Supplementary file 2 — Supplementary Information [file 41538_2023_188_MOESM2_ESM.pdf]

Supplementary Material

Reference proteomes of five wheat species as starting point for future design of cultivars with lower allergenic potential

Muhammad Afzal[1]†, Malte Sielaff[2]†, Ute Distler[2], Detlef Schuppan[3,4]‡, Stefan Tenzer[2]‡ and C. Friedrich H. Longin[1]*‡

[1]State Plant Breeding Institute, University of Hohenheim, Fruwirthstr. 21, 70599 Stuttgart, Germany

[2]Institute for Immunology and Research Center for Immune Therapy (FZI), University Medical Center of the Johannes Gutenberg University Mainz, Langenbeckstr. 1, 55131 Mainz, Germany

[3]Institute of Translational Immunology and Research Center for Immune Therapy (FZI), University Medical Center of the Johannes Gutenberg University Mainz, Langenbeckstr. 1, 55131 Mainz, Germany

[4]Division of Gastroenterology, Beth Israel Deaconess Medical Center, Harvard Medical School, 330 Brookline Avenue, Boston, MA 02215, USA

†These authors contributed equally to this work.

‡These authors share senior authorship.

*** Correspondence:**

C. Friedrich H. Longin

Email: friedrich.longin@uni-hohenheim.de

Supplementary Figures

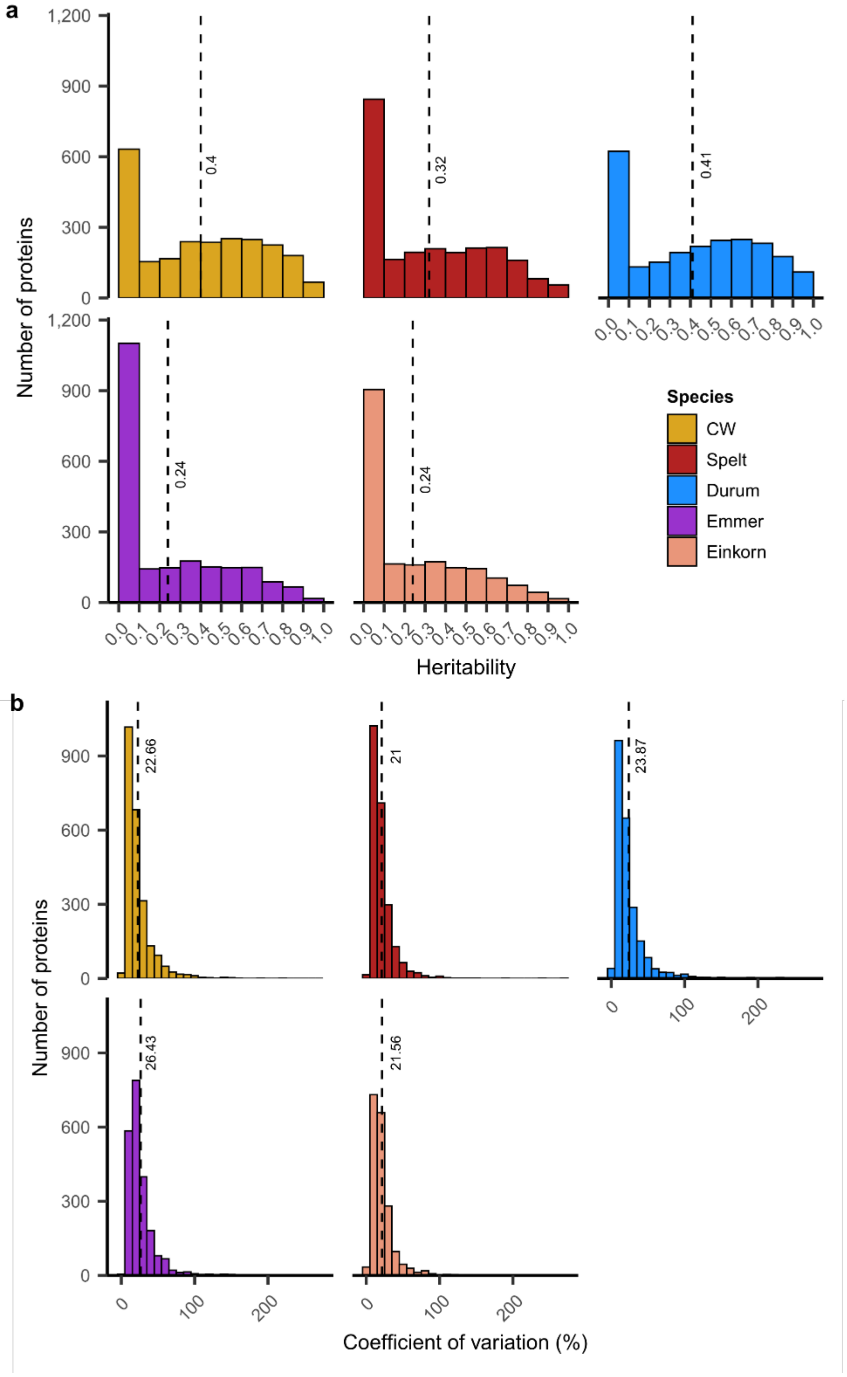

Supplementary Figure 1. Histograms to present **(a)** heritability of proteins of each of the five species. The dashed line and number next to it represent the mean value of the heritability across all proteins of a species from group J in Figure 1a for which the random and fixed statistical models converged, and **(b)** coefficient of variation (CV) of proteins of wheat species. The dashed line and number next to it represent the mean value of the CV across all proteins of a species from group J in Fig. 1a for which the random and fixed statistical models converged. CW, common wheat.

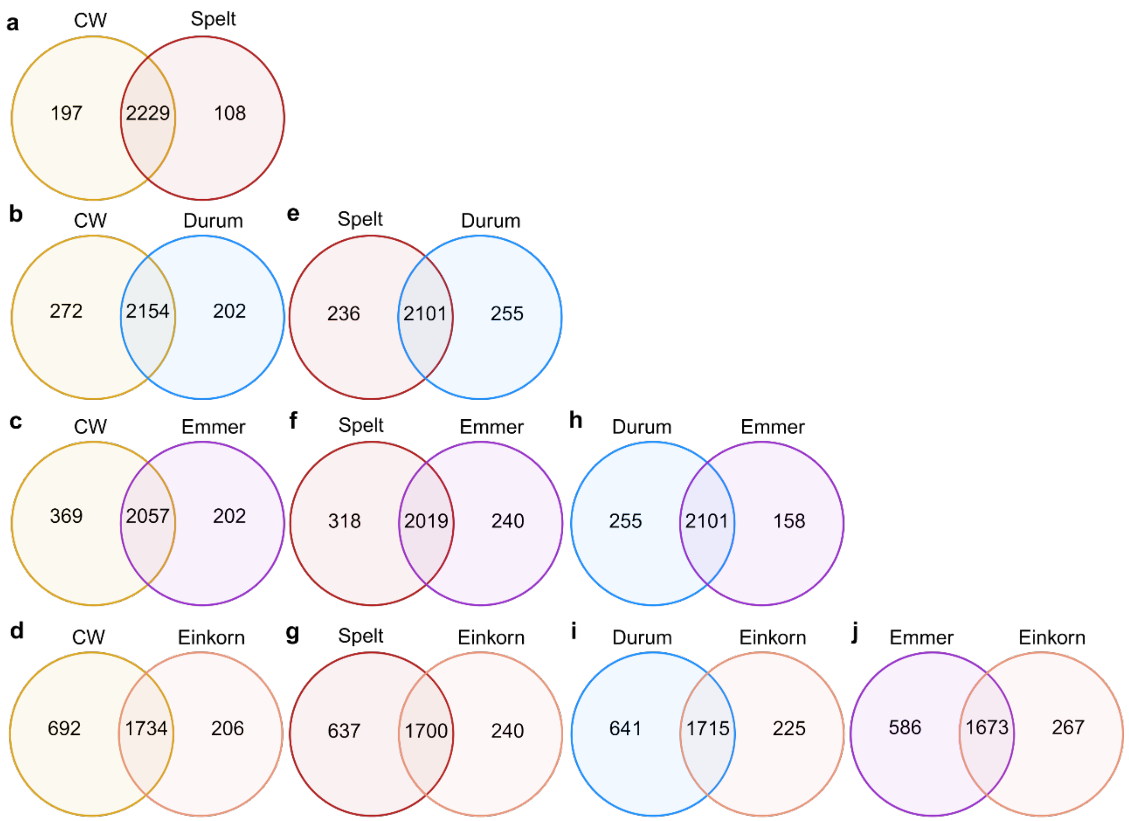

Supplementary Figure 2. Venn diagrams to visualize the number of proteins that are unique or common between different pairs of species **(a-j)**. The proteins belonging to group J in Fig. 1a were used to make the Venn diagrams. CW, common wheat.

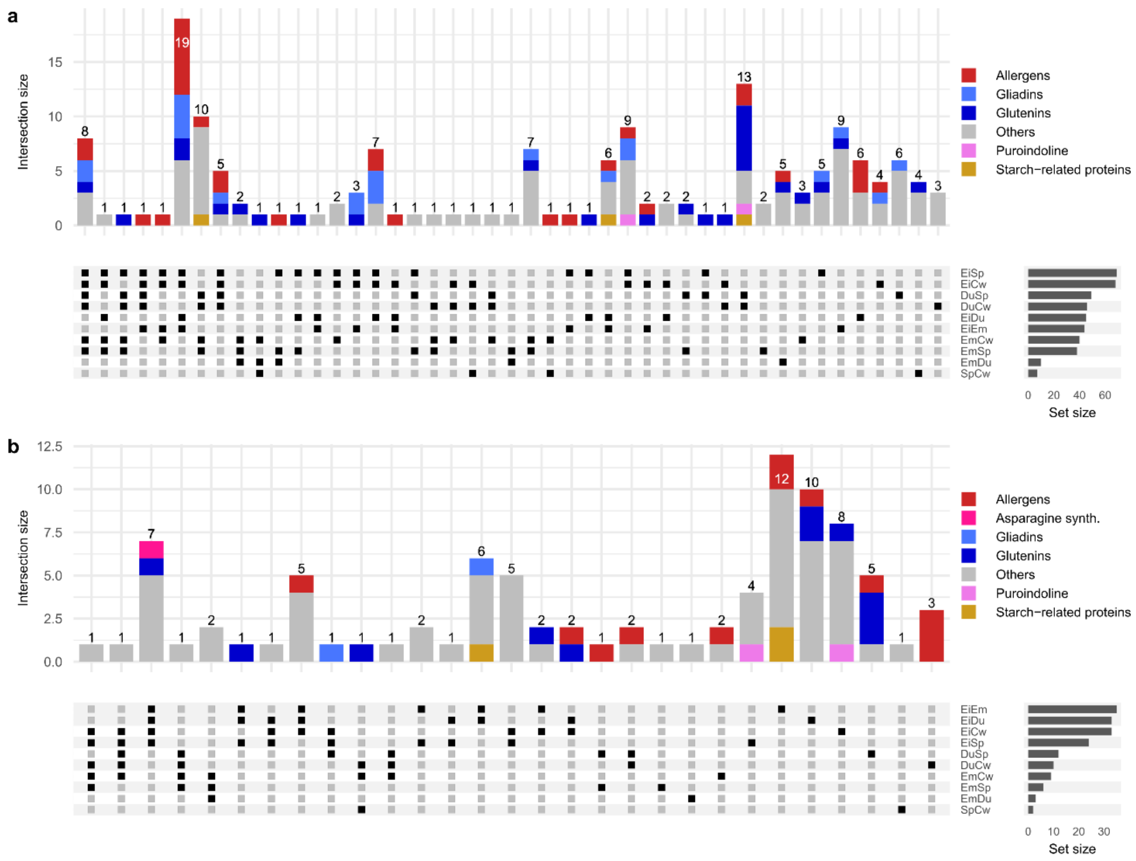

Supplementary Figure 3. Upset plots for **(a)** downregulated, and **(b)** upregulated proteins between different pairs of wheat species. The bar plot shows the number of down- or up-regulated proteins with the dark-colored squares indicating the pairwise species comparisons for which these protein-changes were observed. In the pairwise comparison, the first name is compared with the second, e.g., *EiSp* in (a) means that proteins were downregulated in einkorn compared to spelt. Cw, common wheat; Sp, spelt; Du, durum; Em, emmer; Ei, einkorn.

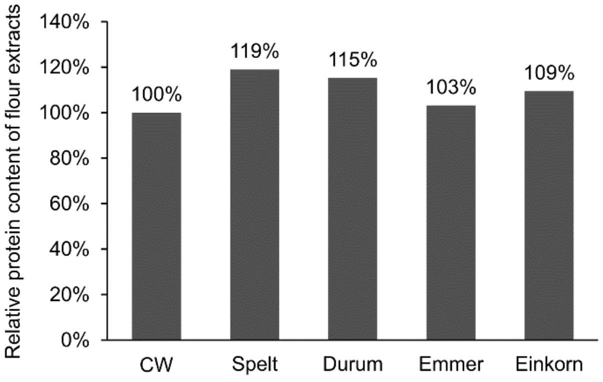

Supplementary Figure 4. Relative protein content of extracts from species-specific flour mixtures as determined by colorimetric protein assay. CW, common wheat.

Legends for Supplementary Tables

Supplementary Table 1 (separate file). Potential candidate proteins for future research and breeding. These proteins were filtered by applying a stringent criterion within each species, i.e., heritability >0.50, missing data ≤20%, detected in all environments in ≥50% cultivars and in at least 2 of 3 environments in ≥80% cultivars.

Supplementary Table 2 (separate file). Results of the t-test for all identified proteins between all possible pairs of species.

Supplementary Table 3 (separate file). Cultivars of five wheat species.

Supplementary Table 4 (separate file). Allergenic proteins.
